# Assessing the impact of local context and priorities regarding domestic disease outbreaks and imported risk on early pandemic response: Cross-continental comparisons

**DOI:** 10.3389/fpubh.2023.1147768

**Published:** 2023-03-23

**Authors:** Fei-Ying Kuo, Tazi-Hung Wen

**Affiliations:** Department of Geography, National Taiwan University, Taipei, Taiwan

**Keywords:** non-pharmaceutical interventions, domestic disease outbreaks, imported risk, stringency index, governmental containment measures

## Abstract

**Introduction:**

Containment and closure policies are effective measures used in the early stages of a highly transmissible global pandemic such as COVID-19 to mitigate the spread and reduce transmissions. However, these policies can have negative impacts on the economy and personal freedom. Governments must carefully consider the necessity of increasing their stringency. Local contexts and priorities regarding domestic disease outbreaks and the risk of imported cases from other countries may vary among different countries, and could influence the decision to increase containment measures. Thus, this study aimed to differentiate the impacts of these affecting factors on the stringency of governmental containment measures through cross-continental comparisons.

**Methods:**

This study utilized a zero/one inflated beta (ZOIB) regression model to investigate how domestic epidemic, imported risk, and local context affect government responses to a pandemic. We used a country’s weekly confirmed case and death numbers as a measure of its domestic threat. The imported risk was measured using a combination of weekly new cases in each country and the air passenger traffic between countries.

**Results:**

The findings indicate that domestic case numbers are a primary concern for governments when deciding to increase policy stringency. Countries with higher development levels tend to implement stricter policies as they can better handle the negative impacts. Additionally, there is an interaction between case numbers and development level, with countries at the second or third highest development level focusing more on domestic outbreaks than imported risks, while those at the highest level have similar concerns for both.

**Conclusions:**

We concluded that most countries adjust policies’ stringency majorly based on the variation of domestic case number rather than the other pandemic factors and the countries with a high development level tend to implement strict policies since their socio-economical condition could afford such policies. These insights can aid policymakers in improving containment and closure policies for future pandemics.

## 1. Introduction

At the beginning of an outbreak of a highly transmissible infectious disease such as COVID-19, the primary focus is on containing its spread through measures such as city lockdowns while effective vaccines or treatments are still in development (1, 2). Previous studies have shown that these containment measures can be effective in slowing the spread of the disease and reducing transmission (3, 4). However, it is also acknowledged that these containment measures can have significant negative effects on the economy and personal freedom. The relationship between the progression of the pandemic and government policy is complex and not fully understood. Some governments may adopt a less restrictive approach at the early stage of an outbreak, for example, Italy aimed to increase herd immunity by implementing less restrictive policies (5, 6). On the other hand, some countries like Taiwan and Singapore have implemented strict containment measures as soon as the first local cases were reported (7, 8). The variation in response can be attributed to a variety of factors such as the country’s level of development, political structure, and population compliance with measures (9). Understanding how containment measures change in response to the progression of the pandemic can provide insight into how governments respond to the threat to public health in the early stages of a pandemic.

Governments must take into account both domestic outbreaks and the risk of imported cases from other countries when responding to pandemics, as the latter can often trigger the former (10). The imported risk of an infectious disease is typically caused by frequent travel from infected countries (11). To reduce this risk during the early stages of a pandemic, the main approach is to isolate infected travelers through border quarantine measures to prevent transmission to local residents. This is because large-scale travel restrictions and border closures can have a large negative economic impact on society (12). However, some infected passengers may mistakenly pass the symptom-based screening, a widely used measure in airports in the initial stage of the COVID-19 pandemic (13); the imported risk, thus, can still pose a direct threat to the local population (14). To mitigate this risk, governments may implement local containment measures such as closing public places or canceling public gatherings to reduce the impact of imported cases (14). The stringency of policies in a country is therefore determined by a combination of both domestic outbreaks and imported cases. Understanding the separate effects of each can help governments make informed decisions when developing epidemic prevention strategies.

A country’s local context, including factors such as political institutions, education levels, and socioeconomic conditions, significantly influences the government’s approach to managing pandemics. These factors can impact a country’s ability and willingness to implement containment measures, as well as shape how effectively such measures are followed by the public. For instance, countries with strong political systems and infrastructures may be able to implement large-scale city lockdowns more quickly (15). On the other hand, countries with robust social welfare systems may be able to better support citizens during lockdowns, which may increase public compliance with containment measures (16, 17). Additionally, populations with high levels of education may have a greater understanding of the risks posed by a pandemic and may be more likely to comply with containment measures (18).

Therefore, the objective of this study is to differentiate the impacts of local context and priorities regarding domestic outbreaks and imported risks on the stringency of governmental containment measures through cross-continental comparisons. By using a zero–one inflated beta regression model, the study aims to analyze the relationship between local contexts and a country’s response to a pandemic. The insights could provide valuable information for policymakers as they strive to improve containment and closure policies. By differentiating the effects on domestic outbreaks and imported risk on the stringency of governmental containment measures, health authorities can better understand the different challenges and trade-offs involved in managing pandemics and make more informed decisions about how to respond to them.

## 2. Materials and methods

### 2.1. Data

The data used in this study for analyzing the effects of domestic outbreaks and imported risk on the stringency of governmental containment measures were obtained from the Oxford COVID-19 Government Response Tracker (OxCGRT) database (19). This database has a panel data format, which records the daily stringency of 19 containment and closure policies for 186 countries on an ordinal scale, with higher numbers indicating stricter policies. To provide a comprehensive, time-varying measure of the stringency of containment and closure policies for each country, the database offers a stringency index (SI). The SI is calculated by summarizing nine relevant indices, including school closures, workplace closures, cancellation of public events, restrictions on gathering sizes, closure of public transport, stay-at-home requirements, restrictions on internal movement, restrictions on international travel, and public information campaigns. The SI was used as the dependent variable in the statistical model, which is characterized by two properties: it ranges between 0 and 100, with higher values indicating stricter containment and closure policies and it contains many zero values because many countries did not implement any containment and closure policies at the beginning of the pandemic.

### 2.2. Study period and countries

**Figure 1:**
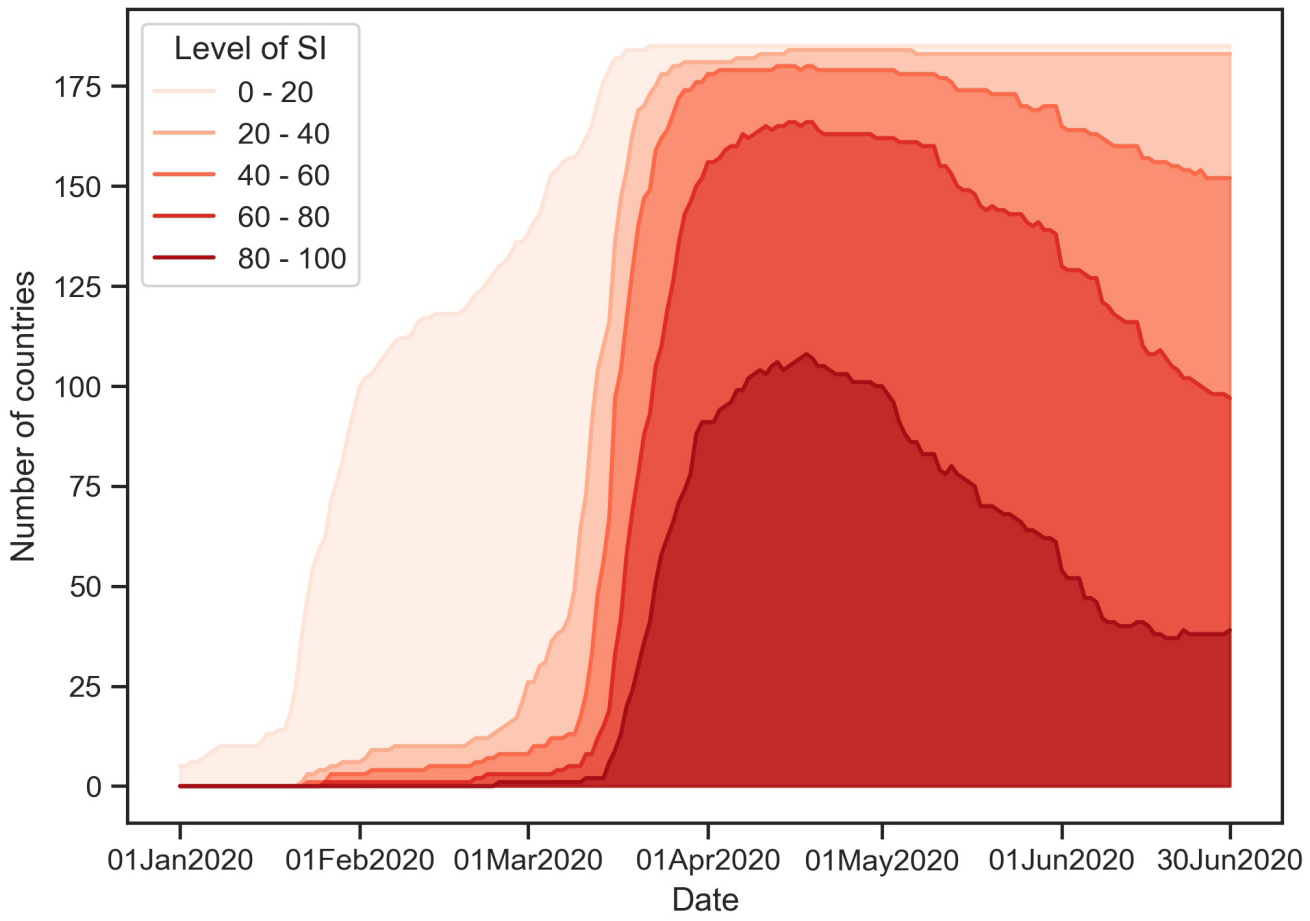
Globally temporal trend of SI level.

The temporal trend of the stringency index (SI) for all countries during the first half of 2020, as shown in Figure 1, illustrates that there was a rapid increase in stringency measures after the World Health Organization (WHO) declared COVID-19 a global pandemic in mid-March, with the peak of stringency reached in mid-April. To capture this trend, the study period was defined as from January 22nd, the date of the first confirmed COVID-19 case, to April 14th, 2020. To account for the delay between the emergence of a pandemic and the implementation of government policies, the analysis unit was set to “week” and the maximum value of SI in each week represents the level of stringency for that week. The study was conducted at the country-level, with each country serving as a separate sample unit and 168 countries out of 186 (90.3%) recorded in the OxCGRT database were included in the analysis.

### 2.3. Independent variables

#### 2.3.1. Domestic threat and imported risk

**Figure 2:**
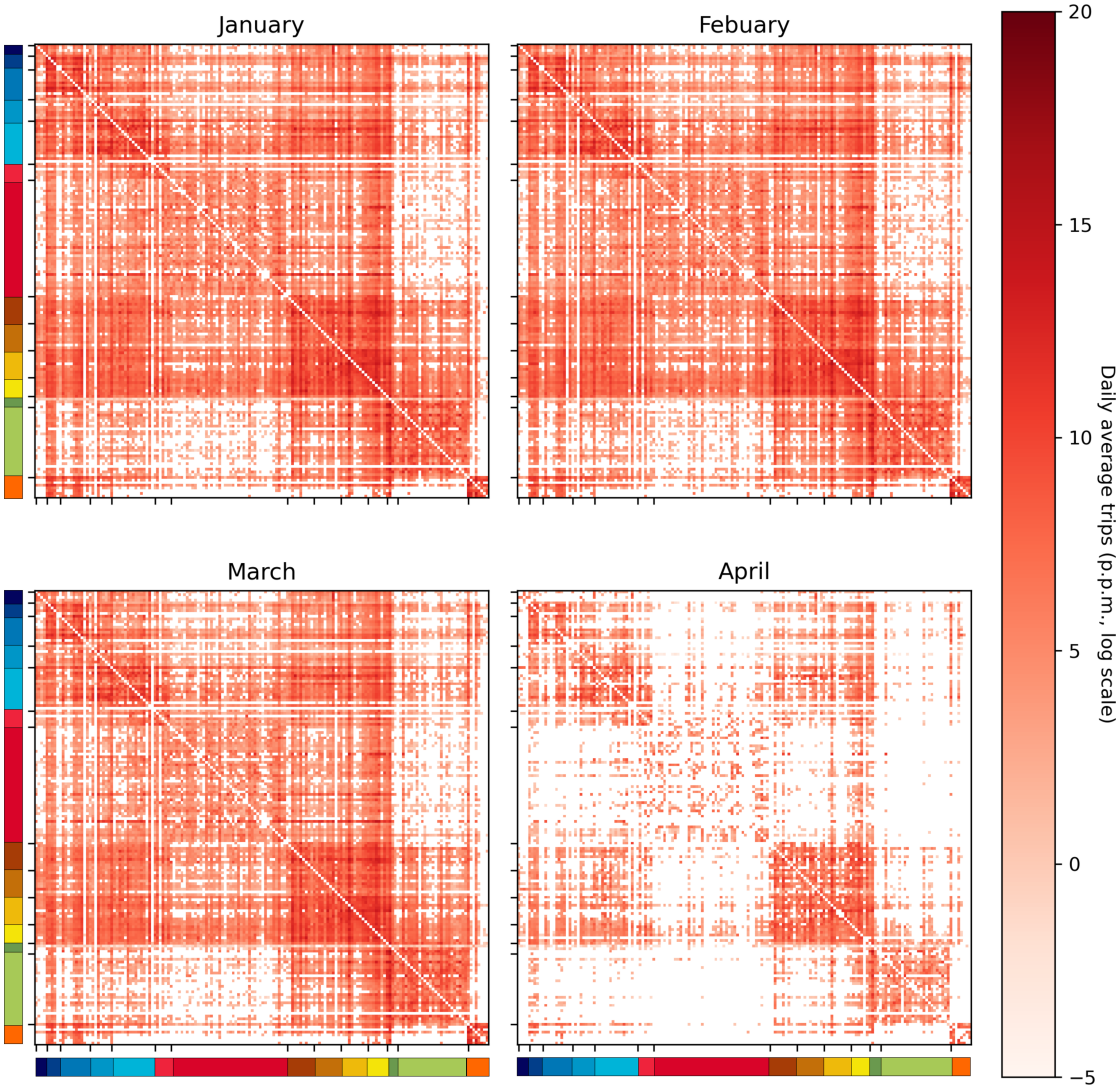
Country-to-country daily average trip number. The color bars on the left and bottom sides indicate the continent to which each country belongs.

The (OxCGRT) database records daily confirmed case and death numbers for various countries, which are sourced from the European Center for Disease Prevention and Control and the Johns Hopkins Coronavirus Research Center (19). Our study uses a country’s weekly confirmed case and death numbers as a measure of its domestic threat. On the other hand, its imported risk is represented by two components: the combination of international travel to the country and the weekly confirmed case and death numbers from other countries. Recent studies also adopted this similar concept to measure the imported risk of COVID-19 pandemic (20). The international travel data used in this study were obtained from the Official Aviation Guide (OAG) (21); it provides the monthly number of air passenger traffic between airports worldwide. Recent studies have also demonstrated the usefulness of the model in reflecting how transnational movements contribute to the global spread of the COVID-19 pandemic (22, 23). We first aggregated the data based on country-to-country relationship and then calculated the daily average number of air passengers between each pair of countries within each month. Figure 2 illustrates this processed movement matrix from January to April 2020. The imported risk caused by overseas cases is evaluated by a linear combination of the number of new cases in other countries and the amount of travel between those countries (Eq. 1).


(Eq. 1) $$C_{1\times n}^{t}.M_{n\times n}^{t}=IRC_{1\times n}^{t}$$

where *C* is a 1 by *n* vector recording the daily number of new cases for each country; *M* is the processed matrix recording international daily average number of air passengers between any two countries; *IRC* represents the daily imported risk of cases; *n* denotes the number of countries, and *t* denotes any specific day during our study period. The weekly imported risk was calculated by summing the daily imported risk over a 7-day period. Similarly, the weekly imported risk caused by overseas deaths was evaluated in the same manner.

#### 2.3.2. Local context

The local context of each country is represented by its 2019 Human Development Index (HDI) and the continent where it locates. The choice to use the HDI from 2019 is to capture a country’s conditions prior to the widespread impact of COVID-19 in 2020. HDI is composed of three sub-indices that measure different aspects of a country, including life expectancy at birth for health, years of schooling for education, and gross national income *per capita* for the economy (24). Although these sub-indices provide detailed differentiation among countries, their significant correlation indicates that HDI alone is sufficient to capture a country’s conditions and differences. Therefore, to facilitate the inclusion of HDI in our model as an independent variable, we standardized it into a *z*-score. The original value ranges for HDI are listed in Table 1 for reference to their corresponding z-score values. Additionally, since countries in the same continent tend to share similar cultural and behavioral characteristics, countries are classified into 14 continents as defined by the United Nations, including Central Asia, East Asia, Southeast Asia, South Asia, West Asia, Eastern Europe, Southern Europe, Western Europe, Northern Europe, Sub-Saharan Africa, North Africa, Latin America, North America, and Oceania. Central Asia was chosen as the baseline for comparison and coefficient estimation since it was the last region where the pandemic spread. Therefore, only 13 dummy variables, excluding Central Asia, were used in the regression model.

**Table 1:** HDI level.

Level	Value range (SD)	Value range (original)
1	Less than −1.00 Std	Less than 0.57
2	−1.00 SD ~ −0.50 SD	0.57–0.65
3	−0.50 SD ~ 0.00 SD	0.65–0.72
4	0.00 SD ~ 0.50 SD	0.72–0.80
5	0.50 SD ~ 1.00 SD	0.80–0.88
6	Greater than 1.00 SD	Greater than 0.88

#### 2.3.3. Interaction effect

We also included interaction terms in our analysis to examine the potential combined effects of pandemic and local context on the SI. To do this, we multiplied each country’s HDI value with its four pandemic-related independent variables (i.e., domestic cases, domestic deaths, imported cases, and imported deaths) and included these interaction terms as additional independent variables in our model. This approach allows us to determine if countries with different HDI levels have a similar relationship between the pandemic and the SI.

#### 2.3.4. Time variable

In order to account for the possibility that the level of SI may have risen over time due to factors such as public opinion or political concern, we included week (Week as variable name) and the square of week (Week2) in our analysis to reflect its linear and non-linear effects, respectively. To avoid estimation bias on a specific variable, all independent variables excluding Region, Week, and Week2 were standardized before being used in the analysis.

### 2.4. Statistical analysis

In order to account for the presence of zero inflation and a fixed range in the dependent variable, SI, this study utilized a zero/one inflated beta (ZOIB) regression model (25). The ZOIB model is specifically designed for dependent variables that have a large number of zero records and a fixed range of values. However, as SI does not exhibit clear one inflation patterns and to improve computational efficiency, the range of SI was scaled to 0–0.99 instead of the standard 0–1. This modification of the ZOIB model combines logistic regression to address zero inflation and beta regression to estimate population mean, as detailed in Eq.2–Eq.6.


(Eq. 2) $$f\left(SI_{it}|\eta \right)=\{\begin{array}{l} p_{it}\\ \left(1-p_{it}\right)Beta\left(\alpha_{it1},\alpha_{it2}\right) \end{array}\;\begin{array}{l} if\;SI_{it}=0\\ if\;SI_{it}\in \left(0,1\right) \end{array}$$

(Eq. 3) $$\mathrm{logit}\left(p_{it}\right)=\beta_{0}^{p}+\overset{J}{\underset{j}{\sum }}\beta_{j}^{p}X_{itj}$$

(Eq. 4) $$\mathrm{logit}\left(\frac{\alpha_{it1}}{\alpha_{it1}+\alpha_{it2}}\right)=\left(\beta_{0}^{\mu }+r_{i}\right)+\overset{J}{\underset{j}{\sum }}\beta_{j}^{\mu }X_{itj}$$

(Eq. 5) $$\log\left(\alpha_{it1}+\alpha_{it2}\right)=\eta$$

(Eq. 6) $$r_{i}\sim N\left(0,\sigma^{-2}\right)$$

where *i* denotes country; *t* denotes each week; *j* denotes the order of independent variables; $p_{it}$ means the probability that $SI_{it}$ is zero; $\alpha_{it1}$ and $\alpha_{it2}$ are shape parameters of the beta distribution. Link functions (Eqs. 3–5) are used to describe the relationship between the main function (Eq. 2) and independent variables ( $X_{itj}$ ). Eq. 3 is the logistic regression to estimate $p_{it}$ , where $\beta_{0}^{p}$ and $\beta_{j}^{p}$ denote the estimated intercept and coefficient of *j*th independent variable, respectively. In Eq. 4, $\alpha_{it1}/\left(\alpha_{it1}+\alpha_{it2}\right)$ represents the mean (μ) of the corresponding beta distribution; $\beta_{0}^{\mu }$ and $\beta_{j}^{\mu }$ denote the estimated intercept and coefficient of *j*th independent variable, respectively; $\;r_{i}$ is the random intercept term for each country. In Eq. 5, η is a constant to fix the relationship between $\alpha_{it1}$ and $\alpha_{it2}$ . Independent variables only influence the probability of zero (Eq. 3) and the mean of a beta distribution (Eq. 4). This model was uses Bayesian inferences for estimating all regression coefficients, and the prior specifications for the parameters in our model are as follows: $\beta \sim N\left(0,10^{-3}\right)$ , $\eta \sim N\left(0,10^{-3}\right)$ , and $\sigma \sim \mathrm{unif}\left(0,20\right)$ .

## 3. Results

### 3.1. Descriptive patterns

**Figure 3:**
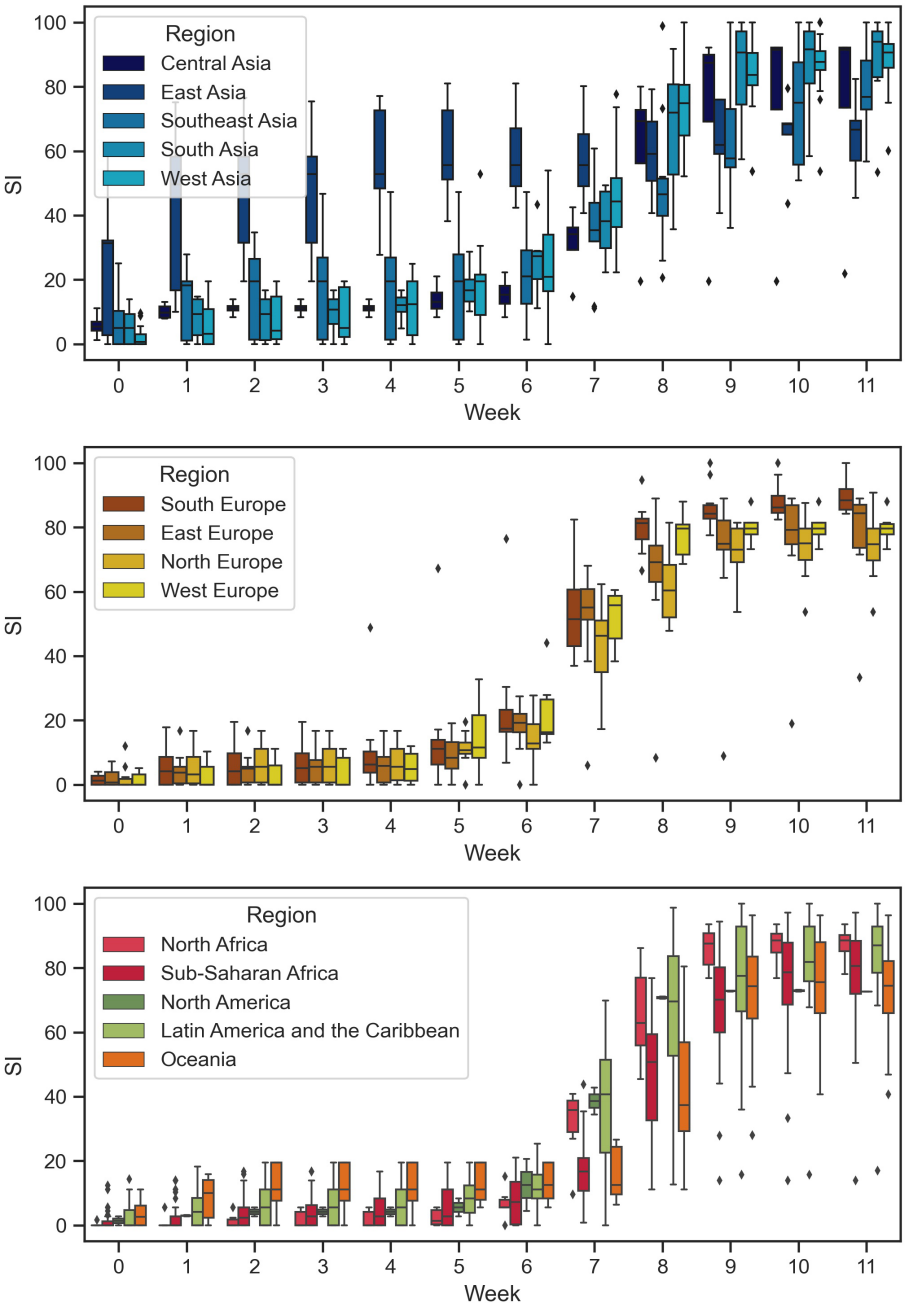
Temporal variations of SI across different regions.

**Figure 4:**
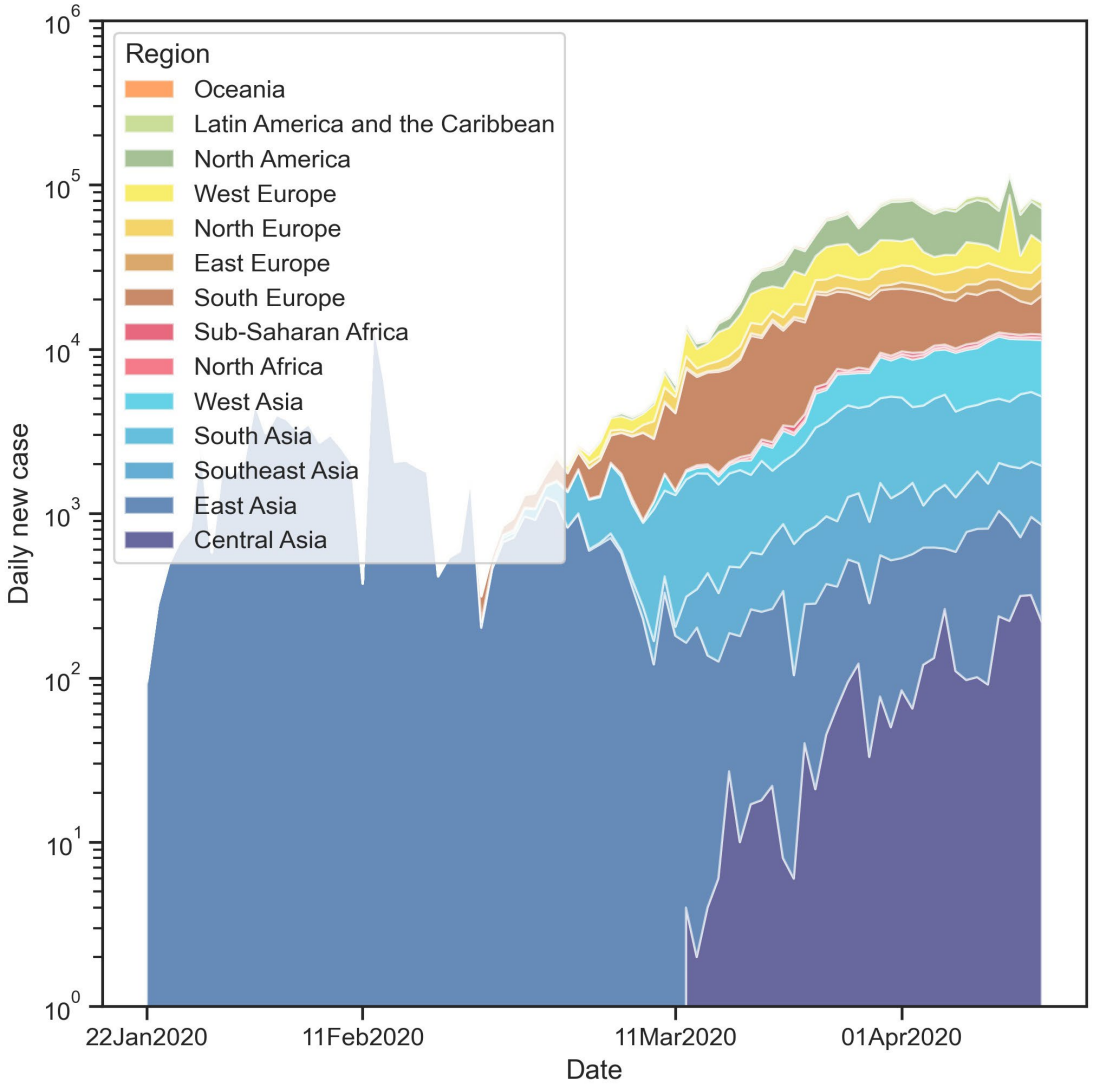
Temporal variation of daily new case number across different regions.

Figure 3 illustrates the variation of the stringency index (SI) within each continent over time during the study period. Some regions, such as East Asia, Latin America, and Oceania, exhibit a higher variation in SI, while the SI in European countries appears to be more consistent. Additionally, countries in East Asia had the highest SI at the beginning, but the SI of other countries gradually increases over time. This pattern could be related to the development of the pandemic (as shown in Figure 4). Since East Asia was one of the initial epicenters of the pandemic, high confirmed case numbers in the region could have prompted local governments to implement stricter containment and closure policies. In contrast, since the pandemic spread to other regions later, their SI levels were not as strict as those in East Asia in the beginning.

### 3.2. Main effects

**Figure 5:**
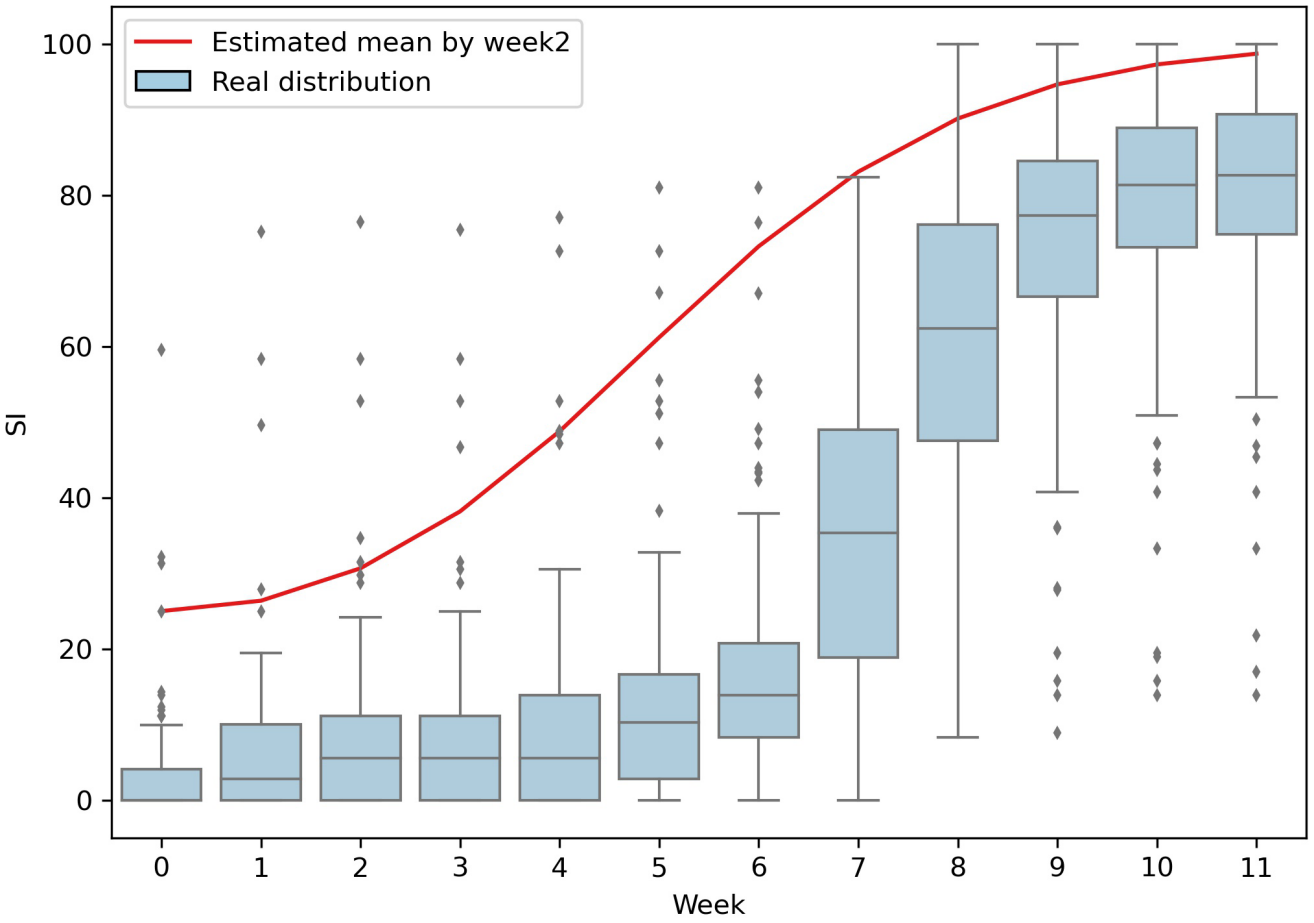
Comparing the estimated SI by only Week2 and the real distribution of SI.

Tables 2, 3 present the results of the ZOIB model. It is found that the variable Week does not have a significant influence on the mean of the beta distribution (*μ*) or the probability for SI remaining at zero (*p* in Eq. 3). However, the variable Week2 does have an effect, suggesting that there is a non-linear time effect on the SI, even when controlling for other dependent variables. This is also visualized in Figure 5, which illustrates the shape of the estimated SI curve and its similarity to the temporal trend of the median of real SI. Therefore, the variable Week2 captures an overall pattern of the SI.

**Table 2:** ZOIB model result.

	Variable name	Mean	2.5% quantile	97.5% quantile	Significance
*μ*	Intercept	−2.470340	−3.093740	−1.947260	*
	Week	0.020200	−0.025060	0.073030	-
	Week2	0.036250	0.031740	0.040660	*
	Domestic case	0.565880	0.134200	0.944230	*
	Domestic death	0.037270	−0.476990	0.607030	-
	Imported case	0.035140	−0.098900	0.142970	-
	Imported death	0.072600	−0.050710	0.214460	-
	HDI	0.189380	0.031140	0.351490	*
	HDI*Domestic case	−0.365810	−0.680080	−0.045370	*
	HDI*Domestic death	−0.107850	−0.561040	0.325960	-
	HDI*Imported case	0.120970	0.031840	0.214950	*
	HDI*Imported death	−0.144110	−0.258600	−0.044170	*
	East Asia	1.102810	0.352360	1.925950	*
	East Europe	−0.190640	−0.853300	0.445900	-
	Latin America	0.103340	−0.471950	0.691550	-
	North Africa	0.092410	−0.645590	0.895820	-
	North America	−0.518080	−1.262810	0.390830	-
	North Europe	−0.317390	−1.008300	0.364840	-
	Oceania	−0.203400	−0.902160	0.478210	-
	Southeast Asia	0.199860	−0.415970	0.861510	-
	South Asia	0.554070	−0.055780	1.287740	-
	South Europe	0.270950	−0.410350	0.925770	-
	Sub-saharan Africa	−0.092690	−0.675370	0.522870	-
	West Asia	0.369840	−0.258310	0.983720	-
	West Europe	−0.047020	−0.748630	0.690910	-

The * symbol indicates a statistically significance of the corresponding independent variable based on Bayesian 95% credible interval.

**Table 3:** ZOIB model result (cont.).

	Variable name	Mean	2.5% quantile	97.5% quantile	Significance
*p*	Intercept	−11.919960	−27.186380	−5.495840	*
	Week	0.015370	−0.197220	0.215310	-
	Week2	−0.072440	−0.103670	−0.040890	*
	Domestic case	−330.773310	−73.682070	3.303080	-
	Domestic death	−8.113760	−37.453830	11.155180	-
	Imported case	−0.240140	−2.258070	2.220230	-
	Imported death	−2.453870	−9.680170	0.618620	-
	HDI	−5.947680	−9.121950	−3.093500	*
	HDI*Domestic case	−15.024300	−33.425410	1.325230	-
	HDI*Domestic death	−27.327470	−51.424700	−0.008560	*
	HDI*Imported case	−0.434910	−1.736060	1.122440	-
	HDI*Imported death	−0.901430	−5.208600	1.095320	-
	East Asia	4.475100	−0.359580	15.995520	-
	East Europe	7.207780	3.198450	18.815630	*
	Latin America	7.170370	3.262060	18.825690	*
	North Africa	8.016240	4.120430	19.379770	*
	North America	5.585760	0.468710	16.721580	*
	North Europe	7.932070	3.901710	19.359670	*
	Oceania	5.478350	1.452900	16.769700	*
	Southeast Asia	6.432820	2.352490	17.485560	*
	South Asia	5.393950	1.427500	16.894720	*
	South Europe	7.364350	3.324690	18.382960	*
	Sub-saharan Africa	6.524530	2.604530	17.943040	*
	West Asia	6.638090	2.674850	18.321020	*
	West Europe	8.447680	4.429400	19.879200	*
*η*		2.168060	2.082100	2.238630	
*σ*		0.243170	0.180810	0.322710	

The * symbol indicates a statistically significance of the corresponding independent variable based on Bayesian 95% credible interval.

Imported risk (both cases and deaths) and domestic deaths are found not to be related to both *μ* and *p*. Only the variable of domestic cases is positively correlated with *μ*, indicating that the weekly number of new domestic cases may be the primary concern for governments to intensify the stringency of containment and closure policies.

Human Development Index is positively correlated with both *μ* and *p*. The estimated coefficients indicate that countries with a higher HDI are more likely to take actions (lower probability for SI remaining at zero) and to increase the stringency of the policies (higher *μ*) resulting in generally higher SI in high-HDI countries.

Finally, it is found that there are clear differences among regions when compared to Central Asia. Only East Asia has a significant positive correlation with *μ*, indicating that countries in this region are more likely to raise their SI. On the other hand, all other regions except East Asia are positively correlated with *p*, meaning that countries in these regions are more likely to wait and monitor the development of the pandemic in its early stages, rather than implementing containment and closure policies.

### 3.3. Interaction effects

**Figure 6:**
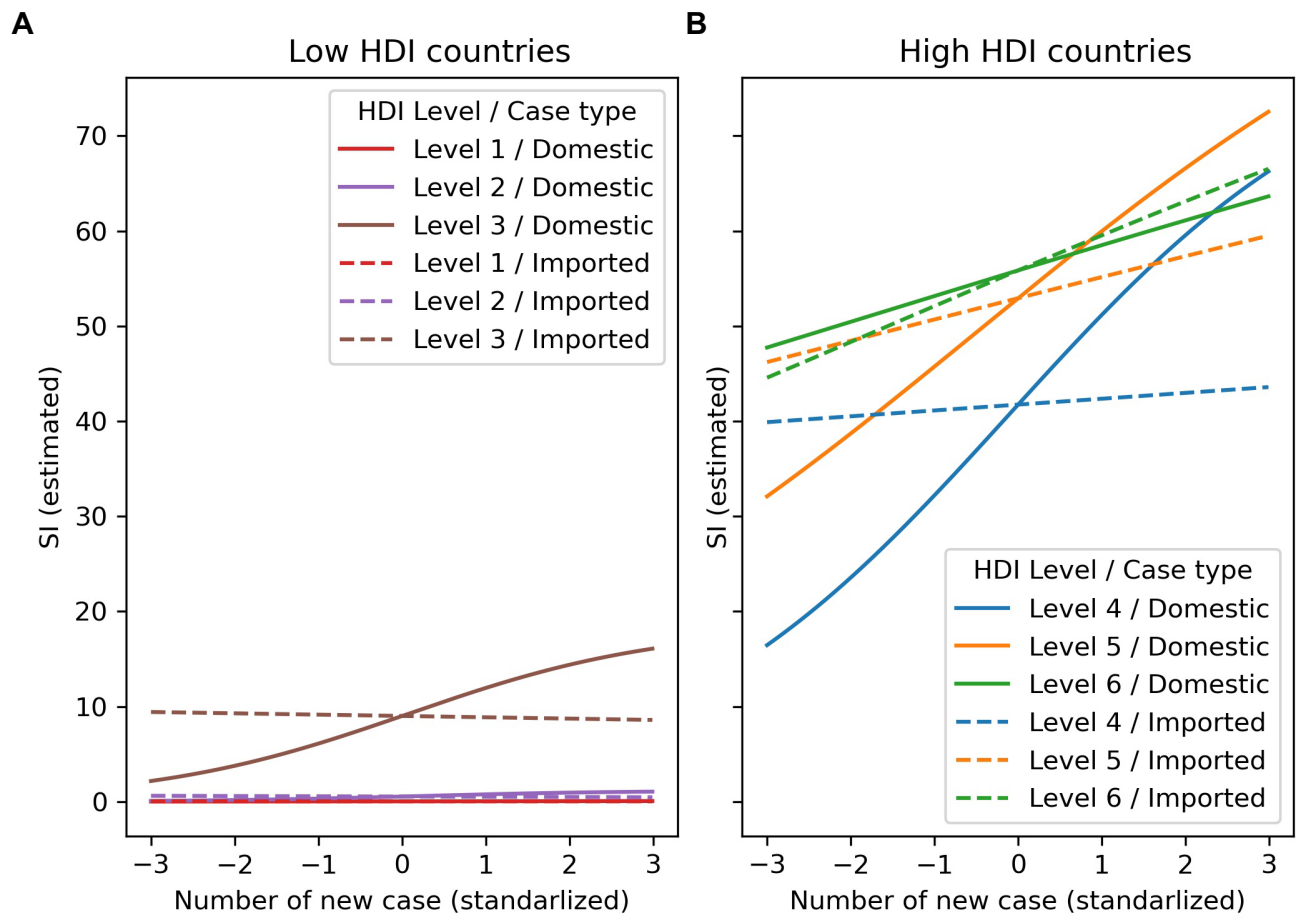
Interaction effect composed of HDI level and domestic disease outbreak or imported risk.

The results of our analysis also show that the interactions between the pandemic and local context have significant impacts on SI level. Specifically, we found that interactions between HDI and domestic case, and imported death have a negative correlation with the mean of the beta distribution (*μ*), while the interaction between HDI and imported case is positively correlated with *μ*. In contrast, only the interaction between HDI and domestic death has a negative correlation with the probability of SI remaining at zero (*p*). Furthermore, the combination of HDI with domestic case is vital for understanding the correlation between weekly new case numbers and SI. Figure 6 illustrates this combination; it shows that while in countries with low HDI, the increase of weekly new cases does not affect SI dramatically. However, in countries with high HDI, the increase of weekly new cases leads to an increase in SI.

Additionally, when examining the effects of domestic cases and imported cases on SI in high-HDI countries, we found that for countries with HDI levels 4 and 5, imported cases are usually a concern for SI, even when their values are low. However, their impact on rising SI is weaker than the impact of an increase in weekly new domestic cases. In other words, when the weekly new numbers of both domestic cases and imported cases are high, the former has a greater impact on SI level. On the other hand, countries with the highest HDI level (level 6) have a reverse pattern, but the difference between domestic and imported cases is not as clear as in the other two high HDI levels (level 4 and 5). Therefore, countries belonging to the highest HDI level may be equally concerned with both types of cases when adjusting SI level.

## 4. Discussion

Our study used a ZOIB model to investigate how domestic epidemic, imported risk, and local context affect government responses to a pandemic. The imported risk was measured using a combination of weekly new cases in each country and the air passenger traffic between countries. The results indicate that the number of weekly new domestic cases is the primary concern for governments when making decisions about increasing the stringency of containment and closure policies. Additionally, the study found that countries with higher HDI values tend to implement stricter policies than those with lower HDI values. Furthermore, countries in East Asia were found to be more likely to take action and quickly raise the stringency of policies. There is also an interaction effect between HDI and weekly new cases. Countries with HDI levels 4 and 5 are more affected by an increase in domestic cases than imported cases, whereas countries with the highest HDI level (level 6) are similarly affected by both types of cases when making policy adjustments.

In this study, it was found that among the nine original indices that make up the stringency index (SI), only “restrictions on international movements” is related to the risk of disease importation. This composition of the index means that SI mainly measures the stringency of local interventions within a country, leading to a lack of correlation between SI and imported risk (both cases and deaths) when analyzing single effects. Furthermore, it was observed that domestic deaths were not correlated with SI when compared to domestic cases. While both domestic cases and deaths can reflect the severity of a country’s domestic epidemic, deaths caused by an infectious disease are typically considered a lagging indicator (26). This means that the rise in deaths reflects the situation several time steps ago and not the current situation of an epidemic. This property of deaths makes it an inappropriate source of information for decision-making in formulating policies against a pandemic, particularly when quick response and reaction are necessary.

The HDI is a measure of a country’s overall development level. Our results indicate that developed countries tend to respond more aggressively to a global pandemic than under-developed countries. Developed countries typically have higher levels of public education, better national health conditions, and stronger social-economic conditions. These factors allow education campaigns about a pandemic to be more effective in making the population aware of the potential loss of life or economic impact if no containment and closure policies are implemented (18). They also enable developed countries to prepare adequate medical and human resources to quickly respond to an outbreak (27). Furthermore, these factors ensure that developed countries can provide financial support to the population, increasing their willingness to comply with intervention policies (28). Thus, developed countries generally have better conditions to implement intervention policies. Benítez et al. (29) found that a country’s socioeconomic condition influences its government’s response effectiveness to the pandemic (29). This supports the idea that low HDI level and poor socioeconomic status in African regions increase their vulnerability to the COVID-19 pandemic (30).

Our findings indicate that high HDI countries may not respond consistently to various types of pandemic threats. Countries with the highest HDI level (level 6), such as the United States, United Kingdom, and Japan, typically have global commercial centers which increases their risk for disease importation (31). This leads national governments in these countries to prioritize both imported and domestic risks (11). In contrast, countries with lower HDI levels (levels 4 and 5) typically do not have global economic centers and may not prioritize imported risks as highly. This also allows them to focus more resources on addressing domestic epidemics, which could account for a steep rise in domestic cases. For example, a long delay in air travel restriction happened in Brazil (HDI level 4) compared to its domestic preventive approaches (32). However, it is important to note that not all countries in HDI levels 4 and 5 disregard imported risks. For instance, Mongolia and Malaysia have taken steps to prevent disease importation before recommendations from the WHO (33, 34). As a general tendency, countries with HDI levels 4 and 5 prioritize addressing domestic threats over imported risks, though some may also take measures to prevent disease importation. Countries with HDI level 6 tend to prioritize both types of pandemics equally.

Our modeling results indicate that only countries in East Asia had active responses to the COVID-19 pandemic. However, this outcome is primarily a result of their geographical proximity to the initial locations of the pandemic, which resulted in greater transnational movement and a higher number of early cases. This proximity has also been observed in previous global pandemics such as SARS in 2003 and influenza A/H1N1 in 2009 (35). As the COVID-19 pandemic initially occurred in China and our study period only covered the first 12 weeks, this proximity led to a concentration of cases in East Asia (as illustrated in Figure 4), necessitating active responses from these countries (36). It is important to note that these results do not suggest that countries in East Asia are inherently more active in responding to pandemics than countries in other regions. Additionally, 13 region dummy variables were employed as control variables to counteract the geographical proximity effect and prevent any potential bias in estimation results of other independent variables.

This study has several limitations. The first is that public opinion and willingness to comply with containment and closure policies may also impact a government’s decision to intensify such policies (37, 38). Although our regression model does not include public opinion as a direct independent variable, the variables “Week” and “Week2” may capture some aspect of this influence. Further studies that include public opinion as a direct variable would be beneficial in understanding this relationship. Another limitation is that this study does not take into account time lag effects. Governments often need some time to formulate and prepare policies after a pandemic enters a new serious phase, and the increase in case numbers may not immediately result in an increase in policy stringency (39). Time lag effects are an important consideration in modeling, but this study’s time unit of analysis is on a weekly basis which may reduce the bias caused by this effect. Last but not least, transnational movements by sea and land transportation were not included in the measurement of imported risk which may lead to underestimating the interaction strength and imported risk between some countries. However, it should be noted that air traffic is the major mode of transnational movement and its absence may not significantly affect the results of the model (40).

## 5. Conclusion

The effect of containment and closure policies on mitigating a global pandemic has been widely investigated, but its inverse direction, how the spread of a pandemic stimulates the rise of policies’ stringency, remains unclear. To deal with this issue, the ZOIB regression model was used to quantify the relationship between the temporal variation of policies’ stringency and the spread of COVID-19 pandemic coupled with 168 countries’ local context. We discovered that most countries adjust policies’ stringency majorly based on the variation of domestic case number rather than the other pandemic factors because most policies are used to control only the spread within territories. Second, countries with a high development level tend to implement strict policies since their socio-economical condition could afford such policies. Moreover, East-Asian counties took actions and raised policies’ stringency quickly in the COVID-19 pandemic because they are geographically adjacent to the origin of the disease. Finally, countries contain global financial centers would concern the imported risk of a pandemic as important as the domestic spread since they encountered considerable amount of transnational movements every day; in contrast, other countries focus majorly on domestic spread. Our findings profiled different responses to a global pandemic among different types of countries, which could be a useful decision support information for every country formulating its appropriate containment and closure policies.

## Data availability statement

Publicly available datasets were analyzed in this study. This data can be found at: https://www.bsg.ox.ac.uk/research/covid-19-government-response-tracker.

## Author contributions

F-YK and T-HW conceived the main conceptual ideas, developed the theory, analyzed the results, and wrote the manuscript. F-YK processed the data and performed the analysis in discussions with T-HW. All authors contributed to the article and approved the submitted version.

## Funding

This research was supported by the grants from the National Science and Technology Council in Taiwan (NSTC 108-2638-H-002-002-MY2 and NSTC 110-2410-H-002-131-MY3).

## Conflict of interest

The authors declare that the research was conducted in the absence of any commercial or financial relationships that could be construed as a potential conflict of interest.

## Publisher’s note

All claims expressed in this article are solely those of the authors and do not necessarily represent those of their affiliated organizations, or those of the publisher, the editors and the reviewers. Any product that may be evaluated in this article, or claim that may be made by its manufacturer, is not guaranteed or endorsed by the publisher.
